# Influence of Baseline User Characteristics and Early Use Patterns (24-Hour) on Long-Term Adherence and Effectiveness of a Web-Based Weight Loss Randomized Controlled Trial: Latent Profile Analysis

**DOI:** 10.2196/26421

**Published:** 2021-06-03

**Authors:** Andre Q Andrade, Alline Beleigoli, Maria De Fatima Diniz, Antonio Luiz Ribeiro

**Affiliations:** 1 Quality Use of Medicines and Pharmacy Research Centre University of South Australia Adelaide Australia; 2 Flinders Digital Health Research Centre Flinders University Adelaide Australia; 3 Caring Futures Institute Flinders University Adelaide Australia; 4 Adult Health Sciences Post Graduation Course Universidade Federal de Minas Gerais Belo Horizonte Brazil; 5 Centre of Telehealth of the Hospital das Clinicas da UFMG Universidade Federal de Minas Gerais Belo Horizonte Brazil; 6 Faculty of Medicine Universidade Federal de Minas Gerais Belo Horizonte Brazil

**Keywords:** obesity, overweight, web platform, digital health, engagement, latent profile analysis, online interventions, use data, weight loss, weight loss platform

## Abstract

**Background:**

Low adherence to real-world online weight loss interventions reduces long-term efficacy. Baseline characteristics and use patterns are determinants of long-term adherence, but we lack cohesive models to guide how to adapt interventions to users’ needs. We also lack information whether very early use patterns (24 hours) help describe users and predict interventions they would benefit from.

**Objective:**

We aim to understand the impact of users’ baseline characteristics and early (initial 24 hours) use patterns of a web platform for weight loss on user adherence and weight loss in the long term (24 weeks).

**Methods:**

We analyzed data from the POEmaS randomized controlled trial, a study that compared the effectiveness of a weight loss platform with or without coaching and a control approach. Data included baseline behavior and use logs from the initial 24 hours after platform access. Latent profile analysis (LPA) was used to identify classes, and Kruskal-Wallis was used to test whether class membership was associated with long-term (24 weeks) adherence and weight loss.

**Results:**

Among 828 participants assigned to intervention arms, 3 classes were identified through LPA: class 1 (better baseline health habits and high 24-hour platform use); class 2 (better than average health habits, but low 24-hour platform use); class 3 (worse baseline health habits and low 24-hour platform use). Class membership was associated with long-term adherence (*P*<.001), and class 3 members had the lowest adherence. Weight loss was not associated with class membership (*P*=.49), regardless of the intervention arm (platform only or platform + coach). However, class 2 users assigned to platform + coach lost more weight than those assigned to platform only (*P*=.02).

**Conclusions:**

Baseline questionnaires and use data from the first 24 hours after log-in allowed distinguishing classes, which were associated with long-term adherence. This suggests that this classification might be a useful guide to improve adherence and assign interventions to individual users.

**Trial Registration:**

ClinicalTrials.gov NCT03435445; https://clinicaltrials.gov/ct2/show/NCT03435445

**International Registered Report Identifier (IRRID):**

RR2-10.1186/s12889-018-5882-y

## Introduction

More than a third (36%) of adults worldwide are overweight (BMI >25 kg/m^2^) or obese (BMI >30 kg/m^2^) [1], which increases the risk for metabolic, cardiovascular, and neoplastic diseases. Daily behaviors such as excessive energy intake and lack of physical exercise are major determinants of excess weight. The advent of the internet and the ubiquity of digital technologies, such as smartphones, has created an opportunity for behavior change at scale.

Interventions delivered using digital technology (digital health interventions) and, in particular, web-based platforms, are promising tools to behavior change and weight management promotion [2]. However, as demonstrated in systematic reviews, their effect is typically short term [3,4]. Technology adherence, defined as the percentage of users who follow an intervention’s intended use pattern, affects the implementation of large-scale digital health interventions [5]. In many ways, adherence has been the Achilles heel of any health intervention aimed at behavior change. The law of attrition, which states that a considerable proportion of users will stop using eHealth interventions, is one of the few laws that has remained consistent throughout the years of digital health intervention development [6]. There seems to be some agreement as to how we can design for adherence, using concepts such as tailoring and fit and combining personal support with digital functionalities [7]. However, developers face several challenges to formulating personalized digital health interventions, given the individual variation of patient characteristics and motivation to use digital health technology.

Adherence to technology can be explained by environmental, technological, and support variables, as well as individual user demographics and psychological characteristics [8]. User characteristics present intervention designers with two challenges. The first is that different user segments may require different products [9]. By understanding user needs, digital literacy, and habits, interventions can be tailored to users’ needs. Tailoring and personalization [10], which also inherently considers individual characteristics, goals, and previous motivation, have been shown to be more effective than static nonadaptive interventions [2]. The second challenge is the high variability on the adoption, response, and outcomes of digital behavior change interventions across users [11]. With some users benefiting more than others, it appears that preintervention individual characteristics are important determinants of adopting a new behavior [12,13]. Given health apps’ low retention rates after the first few days [14], the capacity to adequately adapt apps based on data available on day 1 (baseline and initial use data) could improve user experiences and potentially improve follow-up.

To meet these challenges and improve understanding about user adherence in weight loss interventions delivered through web platforms, we will perform subanalysis of the POEmaS randomized controlled trial (RCT). This provides an opportunity to study users’ baseline characteristics and the impact of these characteristics on adherence and behavior change. Our main goals are to understand the impact of users’ characteristics and the first 24-hour use patterns of a web platform on user adherence within 24 weeks and evaluate whether preintervention characteristics affect the intervention effect within 24 weeks.

## Methods

### Study Design

The Online Platform for Healthy Weight Loss (POEmaS, from the abbreviation in Portuguese) study was a 3-arm (1:1:1), parallel, RCT evaluating the efficacy of a coach-supported online platform for promoting weight loss [15]. The protocol [16] and main results [15] are described in detail elsewhere. The RCT was registered at ClinicalTrials.gov (NCT03435445). This is a substudy of the POEmaS RCT that includes data from the two intervention arms only.

### Setting

The setting was the university community (all campuses) of the Universidade Federal de Minas Gerais, Brazil.

### Recruitment

University students and staff were recruited online using mailing lists and notice boards and through banners and posters across the university campuses reaching current and previous staff, students, and alumni from September to October 2017. They were directed to a website where they received further information about the study, eligibility criteria, and enrollment information.

### Eligibility Criteria

Inclusion criteria for the RCT included age 18 to 60 years, BMI ≥25 kg/m^2^, intention to lose weight through a behavior change program, and internet access. Exclusion criteria included pregnancy, presence of conditions that demand specific dietary or physical activity recommendations (diabetes, heart failure, coronary artery disease, kidney disease, hepatic disease, cancer, phenylketonuria, celiac disease, food allergies, bariatric surgery history), and participation in any other weight loss program at baseline. For this substudy, participants of the comparison (control group) were excluded since their adherence to the intervention was not measured.

### Randomization and Allocation

Those who were eligible were allocated to 1 of 3 study groups using a stratified randomized block design by sex and category of BMI (25 to <30 or ≥30 kg/m^2^) using blocks of variable length (either 3 or 6). Participants then received an email with information about the activities available to the group they were allocated to. The random allocation sequence and algorithm for randomization were developed by a team of information technology specialists who did not participate in the recruitment or assessment processes. Those who did not complete the questionnaires about dietary and physical activity habits during the onboarding process could not proceed to the use of the platform.

### Intervention

The platform-only group was given access to a weight loss program delivered by a web-based platform. The program was based on diet and physical activity guidelines and on the behavior change wheel [17] model comprising a total of 24 weekly sessions (12 weeks of intensive and 12 weeks of maintenance program). Knowledge/empowerment, goal setting, outcomes expectations, self-monitoring, modeling, social support, personalization, and problem solving were the behavior change techniques applied [18]. They were delivered by a range of software functionalities such as short educational readings and videos, graphical and interactive tools, qualitative and quantitative (food diary) dietary monitoring, physical activity self-monitoring tasks, interactive games that created opportunities to invite friends and adopt healthy habits in daily life, and an online social network moderated by physicians and dietitians (Table 1). Personalized feedback on achievements and suggestions of strategies to improve their success in accordance to their individual goals were provided to participants from the fourth week of the intervention. This personalized feedback was generated by a computational algorithm that took into account the goals set by each individual participant, data on habits reported by them in initial questionnaires and through the self-monitoring tools, and patterns of use of the platform (types of functionalities most and least used by each participant) during the first 4 weeks. This platform was adapted from commercial software that had been used for multiple workforce behavior change/wellness interventions in Brazil and is described at length in Beleigoli et al [16].

The platform + coach group followed the same 24-week weight loss program delivered by the platform enhanced by a 12-week initial course of online personalized education and feedback by a dietitian. In addition to the interactions with the platform depicted in Table 1, participants in this group could interact with a dietitian through a private chat forum embedded in the platform. The interactions could be initiated by either side. There was not a limit around the number of interactions that could occur.

**Table 1:** Interactions participants could have with the platform in the two intervention groups.

Category	Interaction
Social	Open the online social network; create or interact with a personal or public post or comment
Self-reporting	Report of a behavior or outcome or entries in the food diary
Content	Read a text or watch a video
Profile	Interaction with profile for personal data visualization

### First 24 Hours of the Intervention

The first 24 hours of the intervention were dedicated to onboarding, including introduction and basic tutorial. Users were informed about which group they were assigned to during onboarding and by email. Both intervention groups (platform and platform + coaching) had access to the same platform and content. Additionally, the platform + coaching group had access to a prerecorded video of the coach welcoming participants to the group. No additional messages were exchanged during the initial 24 hours.

### Data Collection

Baseline data were collected during enrollment and included self-reported anthropometry (weight, height), physical activity and sedentary behavior (short form of the International Physical Activity Questionnaire [19]), health perception (single question derived from the Short Form 36), overall health status (modified from the Short Form Six-Dimension [20]) and exercise stage of change (adapted from Sutton et al [21]); all questionnaires in the validated Brazilian version. We also collected information about health goals, education, and marital status.

To evaluate food content, questions were asked about the total number of daily servings of fruit and vegetables, whole carbohydrates sources, and weekly servings of sweetened beverages and snack foods. This questionnaire was adapted from Molina et al [22]. Eating behaviors were evaluated by including number of days having breakfast, number of daily meals, frequency of take-away food consumption, and eating while watching television. These questions were derived from a dietary guideline tailored to the Brazilian population [23].

For both the food content and eating behavior questionnaires, each question was scored ranging from 1 to 5, with higher scores reflecting higher frequency of recommended habits (eg, consumption of fruits and vegetables and number of days having breakfast) and lower frequency of eating habits that should be avoided (eg, daily consumption of sweetened beverages or eating while watching television). Scores were then averaged to find the final result for each questionnaire.

Additionally, user interactions (clicks and page transitions) were recorded and stored in access logs. Access data were time stamped, categorized according to type of interaction (see Table 1), and filtered to only include data from 24 hours after initial log-in.

Due to intervention design, the initial interaction of control group with the platform was very limited. Additionally, continuous adherence was also limited and, therefore, we excluded this group from the current analysis.

### Outcomes

For this substudy, adherence and weight loss within 24 weeks of the beginning of the intervention were the outcomes of interest. Adherence was defined as the number of distinct days each user logged in to the platform during the first 24 weeks of the intervention. Weight was self-reported by participants at baseline and 24 weeks after the beginning of the intervention. Weight loss was defined as the difference in weight between these two points in time.

### Statistical Analysis

To find the main categories of weight loss intervention users, we analyzed data using latent profile analysis (LPA), a mixture model suited for recovering hidden groups from data [24]. This method is a commonly used cluster method that categorizes individuals into 1 of n groups (or classes), making the findings easier to interpret while losing the least amount of information.

To select the variables used, we initially included all variables describing baseline characteristics (age, eating, and exercise behaviors) and user interactions with the system in 24 hours. Variables that prevented the model from converging or reduced accuracy were removed. The period considered to collect user interactions (24 hours) was selected due to a high number of users (27%) who didn’t return to the app after the first session.

To prevent disproportionate influence from variables with different magnitudes, continuous data were normalized (mean = 0 and standard deviation = 1). Subsequently, we ran the LPA algorithm. To find the best fit, we compared models using different parameters such as the number of possible classes and which variables should be included in the model. We progressively removed variables from the model and ran the algorithm with a growing number of classes, starting at 1 and moving up to 6 classes. The best model was selected using two criteria: Bayesian information criterion (BIC) and highest entropy. The BIC has been shown as a good indicator to select the correct number of classes in mixture models [25]. A lower BIC means a better fit. Entropy is a measure of accuracy (ie, how much each individual belongs to the class it was assigned by the algorithm). High entropy means the individuals were well classified in the groups. Models with similar BIC and entropy were selected based on theoretical plausibility and parsimony.

Finally, we analyzed how classification into the newfound groups predicted future adherence to the intervention and weight loss at 24 weeks (6 months). To test whether the groups are related to long-term adherence and weight loss, we performed the Kruskal-Wallis nonparametric test with a .05 significance level.

Missing weight data at 24 weeks was imputed by fitting logistic and linear regression models with both the predictors and outcome as well as with other variables regarded as important to explain the missing values. This procedure generated 5 complete data sets, which were used to estimate the association between group allocation and primary and secondary outcomes.

We used Python packages Pandas [26] and Scipy [27] for data preprocessing and statistical analysis, respectively. For the LPA, we used the R package tidyLPA [28]. Multiple imputation was performed using SPSS (version 18, IBM Corp).

### Ethics

The study was approved by the ethics committee of the Universidade Federal de Minas Gerais (CAAE: 73545717.5.0000.5149). All participants signed an online informed consent form prior to recruitment.

## Results

Of the 1298 patients enrolled in the POEmaS trial, 828 were randomly assigned to the intervention arms (420 in the platform only group and 408 in the platform + coach group) and included in this analysis. Baseline characteristics can be found in Table 2.

**Figure 1:**
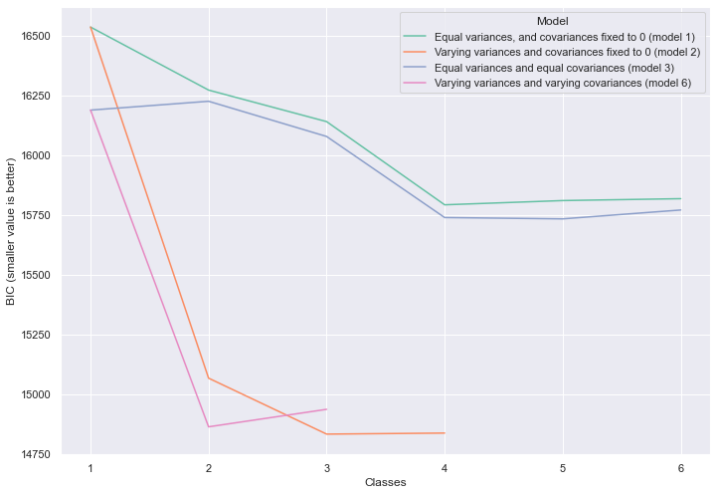
Comparison of different numbers of classes and models as measured by the Bayesian information criterion indicator.

The model with best fit was found using the number of self-report interactions during first 24 hours and scores for 4 questionnaires: the Modified Short Form Six-Dimension, Self-health evaluation (extracted from a single question derived from the Short Form 36), Eating behavior (higher scores means higher frequency of recommended eating habits), and food content (higher scores means frequent consumption of recommended foods such as fruits and vegetables). The optimal number of classes according to the BIC measure was 3, with a BIC 14,833 (see Figure 1).

**Table 2:** Baseline characteristics of the groups included in the analysis^a^.

Characteristic			Platform only group (n=420)		Platform + coach group (n=408)
Weight (kg), mean (95% CI)			83.4 (81.7, 85.0)		82.3 (80.8, 83.7)
Age (years), mean (95% CI)			34.4 (33.4, 35.6)		33.0 (31.9, 34.0)
BMI (kg/m^2^), mean (95% CI)			30.12 (29.67, 30.58)		29.85 (29.44, 30.26)
Female, n (%)			315 (75.0)		319 (78.2)
**Stages of change for physical activity^b^, n (%)**					
	Precontemplation	12 (2.9)		15 (3.7)	
	Contemplation	163 (38.8)		126 (30.9)	
	Preparation	96 (22.9)		94 (23.0)	
	Action	79 (18.8)		91 (22.3)	
	Maintenance	45 (10.7)		43 (10.5)	
	Did not respond	25 (6.0)		39 (9.6)	

^a^Control group participants were excluded from this analysis since they had limited interaction with the platform.

^b^Precontemplation: not intending to engage in physical activity within 6 months; contemplation: intending to engage in physical activity within 6 months; preparation: intending to engage in physical activity within 30 days; action: physically active for less than 6 months; maintenance: physically active for more than 6 months.

**Figure 2:**
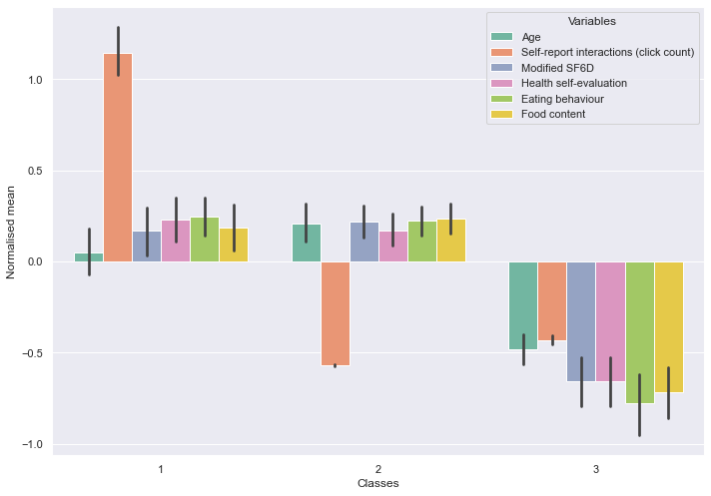
Classes identified in the latent profile analysis as described by the normalized variables.

The 3 distinct groups identified presented significant differences between them (see Figure 2), and the accuracy of the model in correctly classifying individuals was 0.89 as measured by the entropy indicator (entropy for the 2-class model was 0.82 and for the 4-class model was 0.79). Their characteristics are described in Table 3.

There were no significant differences in terms of marital status. Individuals in class 1 had more education years than classes 2 and 3, with 50% graduate students or alumni compared to 40% and 20% in classes 2 and 3, respectively (*P*<.001).

Individuals in class 1 were more likely to select a goal than those in classes 2 and 3, but the choice of goal (lose weight or improve habits or health) was similar in all classes.

At baseline, the classes were significantly different in terms of BMI (*P*=.04), although the difference was small and not clinically important. Users classified as class 1 had a lower BMI, while users in class 3 had a higher BMI than the other classes. Classes also differed according to the number of users assigned to different interventions, with 56% (107/190) of users in class 3 assigned to coaching as compared to 41% (107/260) in class 1.

**Table 3:** Characteristics of users according to latent profile groupings.

Covariates			Class 1 (n=260)		Class 2 (n=378)		Class 3 (n=190)		*P* value	
**Characteristics used to determine classes, mean (SD)**										
	Age (years)	34 (11)		36 (11)		28 (6)		—^a^		
	Food content score	28.7 (4.89)		28.9 (4.22)		24.2 (4.67)		—		
	Eating behavior score	12.5 (1.71)		12.4 (1.59)		10.4 (2.32)		—		
	Self-report interactions click count	12.3 (7.77)		0.2 (0.44)		1.2 (1.26)		—		
	Health self-evaluation	0.64 (0.15)		0.63 (0.14)		0.5 (0.15)		—		
	Modified SF6D^b^	0.73 (0.14)		0.74 (0.12)		0.62 (0.13)		—		
**Other characteristics at baseline**										
	Female, n (%)	200 (76.9)		294 (77.8)		140 (73.7)		.55		
	BMI, mean (SD)	29.46 (4.15)		30.07 (4.63)		30.54 (4.65)		.04		
	Assigned to coaching, n (%)	107 (41.2)		194 (51.3)		107 (56.3)		<.001		
**Stage of change (physical activity) at baseline, % (95% CI)**										<.001
	Precontemplation	3.9 (1.5, 6.2)		2.4 (0.8, 3.9)		4.2 (1.4, 7.1)		—		
	Contemplation	20.0 (15.1, 24.9)		19.8 (15.8, 23.9)		33.2 (26.5, 39.9)		—		
	Preparation	30.8 (25.2, 36.4)		33.1 (28.3, 37.8)		44.2 (37.2, 51.3)		—		
	Action	12.7 (8.7, 16.7)		10.3 (7.3, 13.4)		8.4 (4.5, 12.4)		—		
	Maintenance	29.6 (24.1, 35.2)		20.4 (16.3, 24.4)		8.4 (4.5, 12.4)		—		
	Did not respond	3.1 (1.0, 5.2)		14.0 (10.5, 17.5)		1.6 (0, 3.4)		—		
**Marital status, % (95% CI)**										.27
	Did not respond	38.9 (32.9, 44.8)		64.6 (59.7, 69.4)		58.4 (51.4, 65.4)		—		
	Married	23.1 (18.0, 28.2)		12.7 (9.3, 16.1)		10.5 (6.2, 14.9)		—		
	Divorced	3.9 (1.5, 6.2)		2.7 (1.0, 4.3)		1.58 (0, 3.4)		—		
	Single	33.9 (28.1, 39.6)		19.6 (15.6, 23.6)		29.5 (23.0, 36.0)		—		
	Widowed	0.4 (0, 1.1)		0.5 (0, 1.26)		0 (0, 0)		—		
**Health goal, % (95% CI)**										<.001
	Did not respond	41.5 (35.6, 47.5)		67.5 (62.7, 72.2)		60.5 (53.6, 67.5)		—		
	Lose weight	44.2 (38.2, 50.3)		24.1 (19.8, 28.4)		30.5 (24.0, 37.1)		—		
	Improve habits or health	14.2 (10.0, 18.4)		8.5 (5.7, 11.3)		9.0 (4.9, 13.0)		—		
**Highest educational degree, % (95% CI)**										<.001
	Did not respond	39.6 (33.7, 45.6)		65.1 (60.3, 70.0)		57.9 (50.9, 64.9)		—		
	High school	9.2 (5.7, 12.8)		4.76 (2.6, 6.9)		12.1 (7.5, 16.7)		—		
	University degree	20.8 (15.8, 25.7)		16.1 (12.4, 19.9)		21.6 (15.7, 27.4)		—		
	Postgraduate degree	30.4 (24.8, 36.0)		14.0 (10.5, 17.5)		8.4 (4.5, 12.4)		—		

^a^Not available.

^b^SF6D: Short Form Six-Dimension.

### Associations With Weight Loss and Program Adherence at 24 Weeks

There was a significant difference (*P*<.001) among the classes regarding adherence. Class 1 users were more adherent to the intervention than other users, followed by users in class 2. There was no statistically significant difference in weight loss at 24 weeks (Table 4).

To control for the potential influence of intervention assignment at baseline, we stratified the classes by intervention. The stratification did not change the results. The number of sessions was still significantly different within the platform only group and platform + coach group. Weight loss was not significantly different among users in different classes.

Finally, to investigate whether any particular class of individuals would benefit from different interventions, we stratified participants by class and analyzed their weight change at 24 weeks (see Table 5). Individuals in class 2 who were assigned to the platform + coach intervention had a significantly larger weight loss than those assigned to platform only. The other classes showed no significant difference.

**Table 4:** Number of sessions and weight change at 24 weeks by class.

Outcome			Class 1		Class 2		Class 3		*P* value
Sessions completed in 24 weeks, mean (SD)			9 (19)		6 (12)		4 (6)		<.001
Weight change at 24 weeks (kg), change (SD)			–1.3 (3.6)		–1.2 (3.7)		–1.5 (3.2)		.49
**Platform only**									
	Sessions completed in 24 weeks, mean (SD)	7 (17)		5 (7)		4 (6)		.001	
	Weight change at 24 weeks (kg), change (SD)	–1.2 (3.5)		–0.8 (3.4)		–1.4 (3.3)		.27	
**Platform + coach**									
	Sessions completed in 24 weeks, mean (SD)	12 (22)		7 (15)		4 (5)		<.001	
	Weight change at 24 weeks (kg), change (SD)	–1.3 (3.7)		–1.7 (3.8)		–1.6 (3.2)		.12	

**Table 5:** Differences in weight change at 24 weeks by class.

Outcome		Platform only	Platform + coach	*P* value
**Class 1**				
	Number (%)	153 (59)	107 (41)	—^a^
	Mean weight loss in 24 weeks (kg), mean (SD)	–1.24 (3.48)	–1.34 (3.70)	.93
**Class 2**				
	Number (%)	184 (49)	194 (51)	—
	Mean weight loss in 24 weeks (kg), mean (SD)	–0.80 (3.43)	–1.67 (3.82)	.02
**Class 3**				
	Number (%)	83 (44)	107 (56)	—
	Mean weight loss in 24 weeks (kg), mean (SD)	–1.41 (3.32)	–1.61 (3.18)	.76

^a^Not available.

## Discussion

### Principal Findings

Our data suggest that 3 class groups of users that differ by preintervention characteristics and use within the first 24 hours of the intervention are related to adherence and weight loss outcomes in an RCT testing a web-based platform with and without online coaching for adults with overweight or obesity. The first group (class 1) was composed of users with better eating habits and higher use in the first 24 hours with a lower baseline BMI. The second and largest group (class 2) was balanced in terms of both healthy/unhealthy habits and low/high first 24-hour use. This group was slightly older and had an average BMI similar to that of the whole population. The third and smallest group (class 3) was formed by users with the worst habits and lowest use in 24 hours. This class had higher baseline BMI and younger age than the other groups.

Analyzing the groups’ characteristics at baseline and their longitudinal behavior reveals some insights to help plan future interventions. The first insight is that clustering using 24-hour and baseline data was predictive of higher adherence to the platform 24 weekly sessions. Early identification of the class 2 individuals can help intervention developers adopt different strategies to promote adherence, such as active communication or change in platform content/value proposition. This suggestion is further reinforced by the differences in weight loss between class 2 individuals assigned to platform and platform + coach. The coaching introduces an active component to the intervention that seems better suited to these individuals and resulted in improved weight loss in this study. Enhancing motivation is a key coaching role, so it probably removes some of the effect of preintervention motivation [29].

The second and most important finding, but perhaps least surprising, was that adherence was highest among the class 1 individuals and smallest in class 3. This suggests that this digital health intervention was used by individuals who needed it the least and was dropped by those who would benefit most from it. The path to changing behaviors associated with weight loss depends on a combination of emotional factors, motivation, knowledge, and external factors. Behavior change theories and frameworks, such as the transtheoretical model [13] and the behavior change wheel [17], posit we should consider current individual needs and stages of change to design successful strategies. This is reflected by the higher proportion of users in the action or maintenance phase (physical exercise) in class 1 compared to class 3 individuals. It is also reflected in the larger proportion of individuals choosing health goals, a characteristic of individuals in later phases of change.

This finding reinforces the opportunity for tailoring apps to user current needs. Commonly offered functionalities, such as calorie counters, are better suited to individuals in preparation and action phase. This may explain why studies show a higher baseline BMI and poorer health status in noncompleters [30]. Results also suggest goal setting as a desired functionality for class 1 individuals. This is recommended, given the evidence that goal setting promotes behavior change [31,32]. The 3 groups identified in this study provide an empirical starting point to guide development of different products for different segments.

An innovation in this study was to use the initial 24 hours of user interactions as a latent profile determinant. These interactions may carry information on digital literacy, prior motivation, and whether users immediately perceive the interventions’ value. Early intervention use patterns will vary according to onboarding process and interaction flow. Therefore, it is envisaged that any system collecting interaction data to group users into the 3 classes will require identifying which interaction is most reflective of the initial variable engagement. Finally, in real-world interventions, early interaction may be influenced by factors unrelated to motivation, such as device (mobile vs desktop) or environment where enrollment occurs (work vs home). When known, these factor should be considered to calibrate interaction information.

In this study, the type of intervention had a small but significant influence on the latent profile classification, evidenced by the higher proportion of platform only users in class 1. This was unforeseen, given that data used to discriminate the classes were limited to that collected during the initial 24 hours of the intervention. Additionally, users assigned to different interventions (platform only and platform + coaching) received remarkably similar interventions, with two differences: users in the second group were informed they were assigned to coaching, and they saw a prerecorded video with a welcoming message by the coach. However, our analysis showed that intervention assignment was not the main driver of adherence predicted by the latent profile. Segmented analysis of the platform only and platform + coaching users revealed similar results of differences in adherence between classes.

### Strengths and Limitations

Our understanding about users of digital health interventions aimed at promoting weight loss was facilitated by the very inclusive and pragmatic strategy for recruiting participants. The strategy used for recruiting and the low barrier for joining the intervention mimicked real-life interventions, allowing us to measure how users react outside of controlled trial settings. The use of LPA as a cluster method allowed an improved understanding of users of weight loss interventions. Furthermore, due to the longitudinal nature and multiple repeated measures of outcomes collected, we could analyze the power of the identified classes at baseline in predicting weight loss and adherence at 24 weeks.

Limitations of this study include the self-report nature of questionnaire data, which can carry misclassification biases. Given recruitment happened at a university, the study population may not reflect the general population. This can affect the number of classes identified. Finally, enrollment was an active process that may have selected individuals more likely to benefit from it.

### Implications to Future Research

The intervention planned for the POEmaS project was designed with an active user in mind. In other words, it required users to log in to the platform, report their activities, and interact in the social network. Such interaction was not suitable for the class 3 individuals, evidenced by the large number of users who didn’t return to the intervention. Being able to predict nonadherence allows developers to explore tailoring the intervention, possibly improving intervention effect. The question of how it could be tailored requires further investigation. This study suggests human intervention (coaching) can have a positive effect for class 2 individuals. Additional avenues of investigation include changing the number of active interventions (notifications, emails, or text messages), the content of the messages (focused on contemplation), and understanding the use of gamification techniques aimed at different groups.

This study also highlights the importance of considering the heterogeneity of users when creating and reporting digital health interventions. Any given intervention will affect individuals in different ways based on their baseline habits and motivation. Enrollment strategies may bias the sample (eg, by requiring more active effort before enrollment vs paying participants to complete a study). Setting (clinic, community), barriers to join the intervention (upfront costs, long registration forms, and commitment mechanics), and recruitment method (mailing, targeted communication) all have an impact on selecting the population and, therefore, can influence results. While an additional challenge for creating quality evidence for digital health interventions, careful description of the baseline characteristics and prior motivation of the population groups will create deeper understanding about which intervention is useful for each user.

### Conclusions

Three major groups of weight loss intervention users were identified in a large RCT. Baseline questionnaires and use data from the first 24 hours after log in allowed distinguishing classes, which were highly related to long-term adherence. Individuals classified as class 2 lost more weight when assigned to platform + coach than platform only, suggesting this early classification may be a useful guide to intervention selection. These results encourage efforts for early identification of effect predictors to trigger more effective interventions.

## Abbreviations

BIC: Bayesian information criterion
LPA: latent profile analysis
POEmaS: Online Platform for Healthy Weight Loss (acronym in Portuguese)
RCT: randomized controlled trial
